# Perceptions of adolescents and young adults failing antiretroviral therapy on adherence in Gauteng, South Africa

**DOI:** 10.4102/sajhivmed.v27i1.1759

**Published:** 2026-02-17

**Authors:** Regina M. Molete, Memme G. Makua, Bandile E. Ndlazi

**Affiliations:** 1 Department of Health Studies, College of Human Sciences, University of South Africa, Pretoria, South Africa

**Keywords:** adolescents, HIV, treatment adherence, communication, peer and psychosocial support

## Abstract

**Background:**

The management of HIV among adolescents and young adults (AYAs) living with HIV is often complicated by systemic barriers within healthcare services, particularly communication challenges, inadequate support systems, and other external factors. Understanding these barriers is essential for improving antiretroviral therapy (ART) adherence and overall health outcomes in this population.

**Objectives:**

The study aimed to examine and describe the perceptions of AYAs living with HIV on treatment adherence.

**Method:**

A qualitative approach was applied using semi-structured interviews with AYAs aged 18–24 living with HIV. Twenty AYAs living with both perinatal and horizontally acquired HIV, from four healthcare facilities in Sedibeng District in Gauteng province, with two consecutive viral load results of above 1000 copies/mL identified using the TIER.NET patient management system, were purposively selected and interviewed. Thematic analysis was conducted.

**Results:**

The review identified five key themes characterising the challenges with ART adherence for AYAs. These themes include personal expectations regarding the possibility of HIV cure, and discomfort from side effects, intentional treatment cessation emanating from stigma and disclosure complexities, and healthcare facility challenges, including facility appointments and limited counselling.

**Conclusion:**

The study highlights the need for early identification of barriers to adherence. This should include implementing strategies to enhance accessibility of counselling, and other services to improve adherence and overall wellbeing of AYAs living with HIV.

**What this study adds:** The study provides a detailed examination of the systemic barriers that impede treatment adherence among AYAs with virologic failure on ART. The study highlights and reinforces the idea that timely and comprehensive psychosocial support is essential for AYAs navigating the complexities of living with HIV.

## Introduction

Nearly two thirds of people living with HIV reside in sub-Saharan Africa, and yet are close to achieving the second 95 of the United Nations Programme on HIV/AIDS (UNAIDS) global 95-95-95 targets.^1^ Angola, Mozambique, South Africa, Uganda, and the United Republic of Tanzania have the highest numbers of individuals with HIV who have been tested and diagnosed, but are not on antiretroviral therapy (ART) in sub-Saharan Africa.^1^ By further expanding treatment access and uptake, countries with the largest HIV epidemics could potentially surpass these targets.^1^ Notably, South Africa accounts for approximately 19% of the total global population on ART, with 77% (5.9 million) of people living with HIV in the country currently receiving ART.^1^

The provision of ART has markedly reduced HIV-related morbidity and mortality across populations, especially children and adolescents, and has reduced the risk of HIV transmission.^2^ Despite the benefits of taking antiretrovirals, individuals with suboptimal adherence to ART may not be able to sustain viral suppression. Interruptions in treatment among adolescents and young adults (AYAs) have been associated with rebound viraemia, weakening of the immune system, and increased morbidity and mortality.^3,4^

In sub-Saharan Africa, AIDS represents the leading cause of mortality among AYAs.^5^ Despite the implementation of various strategies aimed at improving adherence to ART to combat HIV, adherence to ART among AYAs continues to pose a significant global challenge.^6^ An examination of the barriers to ART adherence in this population reveals a concerning trend of deteriorating health linked to suboptimal ART compliance, insufficient understanding of the side effects associated with antiretroviral drugs, prevalent misinformation regarding ART, and lifestyle-related challenges faced by AYAs on ART.^7^

Many AYAs find the discomfort caused by treatment side effects to be overwhelming, often leading them to interrupt their treatment. This situation is further exacerbated by socioeconomic factors such as insufficient food, and a lack of transportation money for healthcare facility visits to report these issues.^8,9^ While HIV-related stigma and the complexities of disclosure are pertinent challenges affecting all people with HIV, these challenges are particularly bad for young individuals navigating their self-identity while grappling with the decision to conceal or disclose their HIV status.^10,11^ Those growing up with HIV are often advised by their parents to keep their status confidential because of fears about judgement and intrusive questions, which can increase the likelihood of treatment non-adherence.^12^ Some AYAs may even stop their treatment as an act of retaliation and anger following status disclosure to them by their caregivers, resulting in detrimental effects to their health and treatment outcomes.^13^

Social dynamics can also pose significant challenges for AYAs, especially in shared spaces such as boarding schools or when hosting long-term visitors at home, where they may feel compelled to hide their treatment or find discreet ways to take their treatment.^14,15^ Additionally, many face difficulties in balancing school schedules with healthcare appointments, often prioritising education over health because of the lack of disclosure at school, which limits their ability to seek support from teachers.^16^ The distance to healthcare further complicates matters, as transportation costs can be a barrier to keeping their appointments. In some instances, money for travel may only become available after an appointment has passed, leading to potential conflicts with healthcare staff and resulting in reluctance to visit the healthcare facility while without treatment.^17^

Some scholars have advocated for integrating interventions to address socioeconomic factors such as poverty, stigma, discrimination, and disclosure issues, alongside group-based models for ART adherence.^6,18^ Furthermore, they emphasise the necessity for additional research to elucidate the extent to which these factors impede adherence to ART.^6,19^ This study aimed to explore and describe the barriers to treatment adherence experienced by AYAs living with HIV, to make recommendations for policy and practice.

## Research methods and design

This study employed an exploratory, descriptive, contextual qualitative design, focusing on AYAs living with HIV from March to August 2021. Participants were selected based on their HIV status, unsuppressed viral load, and having missed their appointments.

### Setting

Data were collected in four primary healthcare facilities providing acute and chronic services, and having a high number of patients on ART, with high proportions of unsuppressed viral load, in the Sedibeng District, Gauteng Province. The selection process was further informed by district management, who considered the presence of related studies in the area, and opted to exclude those sites from the study.

### Population and sampling

Participants were purposively selected from a sample frame drawn from the TIER.net report, which identified AYAs with two consecutive unsuppressed viral load results above 1000 copies/mL, on first- or second-line ART regimen at the time of the study. Clinical records were then reviewed to identify those with a history of missed appointments, and who were known to healthcare workers as presenting with treatment adherence challenges. The study included AYAs living with HIV, aged 18–24 years, regardless of the mode of HIV acquisition. This age range was determined to facilitate retrospective data collection, ensuring that participants could articulate their views and experiences, and consent to participation. Considering the nature of the study, the planned sample size was 30 participants; however, saturation was reached with 20 participants during the interviews.

### Data collection

Data were collected through semi-structured interviews with 16 participants, held in a private consulting room within the clinic to ensure privacy. However, because of self-reported COVID-19 infections, four participants were in isolation and opted for telephonic interviews with signed consent forms scanned and submitted. Two trained research assistants, proficient in the local languages, facilitated the interviews. The interview guide was originally developed in English and translated into the participants’ preferred languages, Sotho and Zulu, to ensure comprehension and accurate interpretation. The interview sessions ranged between 30 and 45 min.

The semi-structured interview format employed a partially structured set of questions to elicit participants’ experiences and perspectives. The participants were prompted to discuss the following topics:

- Their expectations while taking ART.
- Positive or negative thoughts that may have influenced their pattern of taking their ART.
- Their thoughts, temptations and reasons for considering discontinuation of ART.
- Their experiences with ART, and its impact on their quality of life.
- Their perceptions of the HIV services provided by the healthcare facility from which they receive care and treatment.

Prompts were used where necessary to facilitate discussion and clarify responses. With participants’ consent, interviews were audio recorded for data capture, and field notes were taken to supplement these data. Member checking was employed to enhance accuracy, by confirming participants’ intended meanings. Confidentiality was maintained by assigning unique identifier codes to interview guides. All data, including field notes and signed consent forms, were scanned and stored in password-encrypted files, while hard copies were securely disposed of. Recorded audio data were uploaded to a computer in an encrypted format, with original recordings deleted from the recording device.

### Data analysis

Individual interviews were audio recorded, and comprehensive field notes were taken during the data collection. The recordings were transcribed verbatim and reviewed for accuracy, to ensure fidelity to participants’ narratives. Following transcription, the data were systematically organised, cross-checked for consistency, and appropriately labelled. A codebook was collaboratively developed by the authors of this article, each analysing a subset of transcripts to identify initial codes and themes. A thematic data analysis approach was applied, following the six steps of thematic data analysis.^20^ In the first step, the researchers familiarised themselves with the data by reviewing the field notes and audio recordings, followed by transcription. The second step involved identifying significant initial codes from the interviews. This initial analysis was followed by the authors’ discussions to reach a consensus on the final themes, enhancing the reliability and validity of the thematic analysis.

Relevant data extracts were organised according to the overarching themes (Table 1), which were reviewed for coherence and identifiable distinctions between themes. Qualitative data were analysed by developing codes based on study objectives and identified themes, which were then categorised to effectively cluster text segments, and to highlight emergent themes related to participants’ ART adherence experiences.

**TABLE 1:** Themes and sub-themes.

Theme	Sub-theme
Personal expectations	Treatment exhaustion Treatment access
Intentional treatment cessation	Disclosure and anger issues Social dynamics
Impaired quality of life	Treatment side effects
Healthcare facility challenges	Healthcare worker’s attitude Long waiting hours and disorganisation
Limited treatment counselling, and support	Difficulty in communicating some challenges with older nurses Limited access to facility support and counselling

### Trustworthiness

To ensure trustworthiness, prolonged engagements were conducted with the participants. Data collection utilised audio recordings to enhance accuracy of the data. During data analysis, data cleaning and member checks were performed. Audio recordings and field notes were compared with the interpreted data to assess, correct errors and seek additional information as needed.

### Ethical considerations

Approval was obtained from the Higher Degrees Committee of the Department of Health Studies at the University of South Africa (reference number: HSHDC/996/2020). Additional ethical clearance was obtained from the Sedibeng District Health Services Research Committee. All participants, aged 18–24, provided written consent for in-person interviews, while verbal consent was obtained for telephonic interviews, with arrangements made for signed consent thereafter. During the selection of participants from TIER.net, measures were implemented to ensure adherence to the *Protection of Personal Information Act 4 of 2013*, stipulating non-disclosure of patient-level data.

## Results

### Participants’ characteristics

A total of 20 young adults comprising 13 women and seven men aged 18–24, living with HIV, were recruited with the assistance of healthcare workers. The participants included four individuals aged 18, five aged 19, four aged 20, one aged 21, two aged 22, two aged 23 and two aged 24. During the individual interviews, some participants opted out of certain questions, exercising their right to decline to respond to questions they found uncomfortable. However, this opt-out did not affect the quality of the study findings, as only two of the 20 participants opted not to respond to the question pertaining to healthcare facility challenges.

### Adolescents and young adults’ personal expectations on HIV treatment

Participants in this study articulated their anticipations and the difficulties encountered with lifelong treatment. This reflects a desire to continue treatment with the hope that it may eventually be discontinued. Additionally, participants detailed the barriers and aspirations associated with being in lifelong therapy. One participant remarked:

‘Taking this treatment with an expectation that it will keep me healthy until there’s a permanent cure.’ (Participant 1, 19-year-old, male)

For some participants, treatment adherence was accompanied by unforeseen challenges that, at times, may have contributed to suboptimal compliance. One participant said:

‘It is very sad to learn that I will have to be on treatment for my entire life. I had hoped that at some point I would be told to stop treatment and be healed. The nurses keep introducing different kinds of treatment, which is confusing at times.’ (Participant 5, 19-year-old, male)

For some participants, personal expectations and perceptions of treatment contributed to a sense of treatment exhaustion, as they anticipated a future moment when they would be advised to discontinue treatment or receive a one-time intervention. The failure to meet these expectations led some participants to view their medication as a form of punishment. This sentiment was expressed by some participants, who stated:

‘I wish for a permanent cure for HIV rather than this life sentence I am living. Being on this medication is really draining to a point where I even forget treatment collection dates and appointments.’ (Participant 14, 18-year-old, female)

‘I wouldn’t say I stopped taking my treatment, but would just hide it, skip a few days and sometimes weeks. I was tired of these pills. They are too big, taste awful and have to be taken every day without a break.’ (Participant 8, 20-year-old, female)

Participants also discussed the impact of restricted access to treatment during the COVID-19 pandemic. One participant stated that:

‘I was on the Central Chronic Medicines Dispensing and Distribution [*CCMDD*] programme used to get my treatment at the pharmacy, which was closed on two occasions due to the COVID-19 pandemic as staff members were in isolation, and I went without treatment for a while.’ (Participant 11, 18-year-old, male)

### Intentional treatment cessation

Participants reported instances of discontinuing, temporarily pausing, or omitting several doses of their treatment for various reasons. One participant remarked:

‘I have in the past stopped taking my treatment a few times, and this was because of my family’s attitude towards me after I had disclosed. I received no support whatsoever from my family. I realised that I had no reason to live, looking at the way I was being treated.’ (Participant 17, 23-year-old, female)

Feelings of anger and internalised stigma prompted some participants to choose to discontinue their treatment. One participant elaborated, stating that:

‘I once stopped taking my treatment as I was angry at my parents when I learned from TV that the pills I am taking are for HIV, and that was when my grandmother told me that I was born with HIV.’ (Participant 2, 19-year-old, female)

The requirement to adhere to a daily medication regimen was perceived as burdensome by some participants, leading to a preference for long-term injectable options. As one female participant expressed:

‘It would be great if I could get treatment that can be taken occasionally, like injectable contraceptives, to avoid having us go to the clinic for collection all the time.’ (Participant 19, 21-year-old, female)

Social dynamics were identified as factors contributing to treatment interruptions, leading some patients to inadvertently discontinue their medication. This was illustrated by a participant who stated:

‘At some point, my dad decided that we were moving back home in [*sic*] Zimbabwe since he had no job at the time. This was an abrupt decision, and he forgot to collect a transfer letter for me, which was then a challenge to get treatment until we arranged for someone to collect and send it. I was forced to stay for a few months without it.’ (Participant 16, 20-year-old, male)

HIV treatment can be particularly perplexing for individuals who acquired the virus perinatally, as they transition from adolescence to adulthood. This confusion often arises as they begin to question their circumstances. One participant remarked:

‘I was told that I contracted HIV during my mother’s pregnancy, hence I was initiated on treatment while still a child. But growing up, I learnt that HIV is sexually transmitted, and at the time, I did not even have a boyfriend. I asked a few of my peers about this kind of HIV, and none of them knew about it and were on such treatment. It is from there that I decided to stop taking it.’ (Participant 1, 19-year-old, female)

### Impaired quality of life: Treatment side effects

Participants believed that adherence to ART would enhance their quality of life. However, they occasionally skipped doses and contemplated discontinuing treatment because of side effects and occasional illnesses. Many expressed that these adverse effects served as significant barriers to their adherence:

‘So far, I have not seen any change since starting this treatment except for discomfort from side effects.’ (Participant 18, 23-year-old, female)

Participants reported experiencing side effects that were perceived as uncomfortable, with some expressing a desire for a treatment option that would not produce side effects and could be administered as once-off medication with a shorter duration such as antibiotics and stopped after completing the course. This sentiment was illustrated by one participant who remarked:

‘It would be great if we could have treatment or therapy that has fewer or no side effects and prevents infections and be taken once a week at least.’ (Participant 8, 20-year-old, female)

Certain side effects reported by participants were noted to be unbearable and experienced frequently. One participant stated:

‘Treatment side effects are a problem every time. An hour after taking my pill, I feel like a zombie and forced to go to bed. Sometimes I feel like I am losing my mind.’ (Participant 3, 24-year-old, female).

While the participants in the study acknowledged and valued the benefits of HIV treatment, it was observed that such treatment is accompanied by a certain level of discomfort for some. One female participant remarked:

‘I really appreciate this treatment despite the nuisance of taking it daily as it makes me drowsy, nauseous and tired at times.’ (Participant 4, 18-year-old, female)

The participants conveyed different sentiments regarding their quality of life, some asserted that the ART had positively impacted their quality of life. According to the participants:

‘I used to be constantly sick, which was really affecting my life and school performance, but since starting treatment, I no longer get sick frequently.’ (Participant 12, 19-year-old, male)

It was also observed that while some participants experienced various ailments before initiating HIV treatment, others reported no noticeable difference in their condition. As one participant stated:

‘I have no idea if this treatment has made any difference in my life. However, I am still alive, maybe that’s the only thing that’s there as a benefit.’ (Participant 9, 20-year-old, female)

For certain participants, it appeared that they observed a change in their health status, as they were no longer experiencing other infections and ailments. One participant remarked:

‘I don’t know whether it’s the [antiretrovirals] that are working or the other meds I was given at the clinic, but I no longer experience the recurring sores or wounds. That has at least got better.’ (Participant 8, 20-year-old, female)

### Healthcare facility challenges

The participants indicated that the attitudes of healthcare providers shifted when clients failed to attend their scheduled appointments. As one participant noted:

‘The nurses at the facility are very good and great when starting treatment, but once you are on treatment for a longer period, their attitude changes. It gets worse when you miss your appointment, you immediately get a new name.’ (Participant 13, 22-year-old, female)

This was also alluded to by the statement from another participant, saying:

‘I understand that nurses are doing their job and want what’s best for us, but they become very rude when you’ve missed your appointment, and that makes it even scarier to go back.’ (Participant 19, 21-year-old, female)

Some participants indicated that the services lacked flexibility and convenience for the youth demographic. Additionally, some expressed that prolonged waiting times served as a deterrent, discouraging them from visiting the facility or adhering to their scheduled appointments. One participant noted:

‘Coming to the facility is a challenge for school-goers because that means missing classes the whole day. It might be a good idea, although it also might expose us to others, to have the school health nurses delivering our treatment at schools and also collect our blood in a safe and private space organised with the principal. In this case, I get to miss a few minutes of classes but also get my clinic services conveniently.’ (Participant 16, 23-year-old, male).

Poor file management was also reported by several participants, as one individual stated:

‘My clinic is good and has good people working there, but their filing system is a mess. My file always gets lost. You even get surprised when it is found without it being a mission.’ (Participant 6, 20-year-old, female).

A limited number of healthcare providers was perceived as a constraint on access to services and the timely delivery of efficient care, as indicated by a participant’s statement:

‘The service here is slow, and you end up spending almost the whole day in the clinic when you have to renew your prescription, although there’s a men’s clinic on the other side, but it is also understaffed, which doesn’t help.’ (Participant 7, 24-year-old, male)

### Limited adolescents and young adults-focused treatment, counselling and support

The generational gap between healthcare providers and AYAs presents several challenges related to communication, guidance, and the comprehension of behavioural dynamics:

‘Most of the nurses are of my mother’s age, and that poses a communication barrier, as I am afraid to ask questions, for I feel like I will be judged.’ (Participant 4, 18-year-old, female)

‘I wish we could be separated from adults so that we can support each other.’ (Participant 5, 19-year-old, male)

Participants reported varied experiences and perspectives regarding the services offered, as well as the accessibility of counselling and support from staff across different facilities. According to the participants:

‘The services I receive in the clinic are good, and I am happy with the clinic staff. The only problem is that at times only one nurse is working, and we must wait longer to get assisted because the same nurse also takes blood from all the patients.’ (Participant 20, 19-year-old, female)

Participants indicated that the facilities lack an adequate number of accessible counsellors to deliver continuous treatment support as needed, resulting in delays when seeking counselling services. One participant noted that:

‘When referred for counselling, one has to wait in another long queue as there is always a shortage of counsellors.’ (Participant 10, 22-year-old, female)

In addition, participants felt that a valid justification was required to consult with the counsellor, as the counsellors are often occupied. One participant remarked:

‘It is not easy to see the counsellor; nurses would refer you only when you have a valid reason.’ (Participant 17, 23-year-old, female)

## Discussion

This qualitative study provides a nuanced exploration and contextual examination of perceptions on treatment adherence among AYAs living with HIV. It explores the narrated experiences of young individuals living with HIV with unsuppressed viral load and some history of missing facility or treatment collection appointments. The selected participants were all active clients receiving ongoing HIV care, and according to data on TIER.net and recorded information, with at least two consecutive unsuppressed viral load results. The findings highlight a complex interplay of personal expectations, social dynamics, systemic barriers, and the impact of healthcare services on treatment adherence.

### Psychological burdens of chronic illness

The participants expressed different expectations for lifelong treatment, with some expressing a desire for a cure or the eventual cessation of treatment. This feeling may explain why participants in a separate study cited ‘feeling better’ as the primary reason they stopped taking ART.^21^ Many expressed disappointment upon realising that ART is lifelong, which contributed to emotional fatigue and treatment interruptions. Treatment fatigue was further exacerbated by pill burden, exposure to other treatments and daily routines, mirroring findings from similar studies where AYAs resorted to ‘drug holidays’ as a result of psychological and physical exhaustion.^21,22,23^ Unmet expectations and psychological burdens associated with chronic illness can significantly impact adherence, particularly when patients perceive treatment as a ‘life sentence’ rather than a potentially life-saving intervention.^24,25^

### Balancing the benefits and burdens in HIV treatment adherence

While many AYAs acknowledged the positive impact of ART on their overall health and quality of life, they also highlighted concerns regarding adverse side effects that hindered their daily functioning. Common complaints included fatigue, nausea, and cognitive impairment, which led some participants to question the efficacy of their treatment. The findings concur with previous studies that have cited complications arising from treatment side effects as a significant factor in poor treatment adherence.^26,27,28,29^ The dichotomy of experiencing both the benefits and burdens of ART reflects the complexity of managing a chronic condition and the need for comprehensive side effect management and counselling.^28^ This highlights the need for continuous peer support, adherence counselling and the establishment of mechanisms for reporting and managing treatment-related adverse events at the healthcare facility level.

Treatment literacy is vital for patients and AYAs, to understand their treatment and what to expect from it. Confusion surrounding treatment changes and the introduction of new generic medications contributed to feelings of frustration and non-adherence in some participants. This confirms the findings from Malawi, which highlighted poor treatment self-efficacy and misinformation as a significant contributor to non-adherence.^30^

### Navigating stigma and support

The findings of this study indicate that treatment interruptions are often linked to social dynamics, particularly family attitudes and stigma. The lack of family support exacerbated feelings of isolation and despair for some participants, leading to medication concealment and non-adherence. Similar findings were reported in a Nigerian study, where family members withheld food and other utilities because of the individual’s HIV status.^31^ HIV status disclosure to family members encountered varying degrees of support, ranging from supportive to rejecting, and emotional distress leading to intentional treatment cessation. Research has shown that stigma and discrimination at home and outside represent a significant barrier to treatment adherence; however, these issues were rarely addressed during counselling sessions.^15,30,32^ In contrast, some AYAs reported that disclosing to some family members proved beneficial, as these relatives provided reminders and encouragement regarding treatment adherence.^26,27^ The effectiveness of family support depends on the involved family member’s maturity as some resort to threatening messaging, and harsh discipline to encourage adherence.^24^ Having a trusted individual or sharing a similar status at home increases the level of support because being the only individual living with HIV in the family was associated with poor support adherence.^31,32^ The study findings also reveal the contribution of internalised stigma to treatment interruption, highlighting the need for enhanced counselling and support. Strategies to enhance adherence should focus on managing status disclosure and medication concealment to optimise treatment adherence.^33^ Addressing stigma and developing strategies to manage internalised and perceived stigma as well as family-enacted stigma is essential among AYAs living with HIV.

### Disclosure and stigma among adolescents and young adults with perinatal HIV

Adolescents and young adults living with perinatally acquired HIV expressed uncertainty regarding their HIV status due to delayed disclosure, which contributed to feelings of mistrust and denial. These adolescents were identified by other studies as less likely to adhere to treatment, primarily due to a lack of understanding of the reasons for taking treatment and a form of denial and rebellion.^27,31^ The internalised stigma emerged as a significant factor, in which participants expressed feelings of anger and resentment towards their circumstances, especially parents, occasionally resulting in treatment cessation. Studies conducted in South Africa and Botswana have similarly found that both internalised and perceived stigma adversely affect ART adherence by influencing the motivation, timing, location and way medication is taken.^33,34^ These findings highlight the critical importance of timely and age-appropriate disclosure for AYAs living with perinatal HIV.

### Impact of external factors

The impact of external factors, particularly the coronavirus disease 2019 (COVID-19) pandemic, further complicated treatment adherence among participants. The findings corroborate those of review studies that identified limited access to facilities as a major barrier to treatment adherence during the COVID-19 period.^35,36^ In some cases, disruption may also contribute to a lack of assessment and management of side effects, which could lead to treatment interruptions.^36^ Additionally, abrupt relocations with parents during the COVID-19 period, often without obtaining the necessary transfer documentation because of loss and change of work, were identified as factors contributing to instances of treatment interruption and eventual non-adherence.

### Healthcare access and communication

The findings of this study indicate significant systemic barriers within healthcare services that impede treatment adherence. This aligns with previous research indicating that inefficient clinic processes, long waiting times, and staff attitudes deter adolescents from accessing healthcare.^26,37^ Furthermore, the negative attitudes of healthcare providers, particularly towards those who missed appointments, further exacerbated the challenges faced by AYAs. Contrary to this finding, a Ugandan study reported excellent healthcare worker attitudes toward AYAs living with HIV.^38^ The challenge of collecting treatment for working and school-going adolescents persists in many healthcare facilities. The findings were similar to studies conducted in other areas, which have identified school schedules as a significant factor contributing to missed healthcare facility appointments and treatment adherence, particularly when adolescents have not disclosed their status to teachers and experience poor teacher-learner interactions.^27,34,37^ Participants indicated that the use of school health services could be beneficial to facilitate treatment deliveries including venepuncture blood collection. This approach could enhance accessibility and adherence to treatment among AYAs.

The generational gap between AYAs and healthcare providers also emerged as a barrier to effective communication and support. The age difference made communication and asking questions difficult for them, and they felt they would be subject to scrutiny. Similar sentiments were echoed by adolescents in other studies, who emphasised the need for friendlier healthcare providers.^15^ Such an environment would facilitate open discussions regarding treatment questions and challenges, including information on viral load suppression.

### Enhancing adherence through counselling and support

Another critical barrier identified in this study was the lack of accessible counselling and support services. Findings from a similar study also allude to suboptimal psycho-social support, with a deficiency in comprehensive information on HIV, adherence and viral load suppression.^32^ Progressive and continuous counselling on living with HIV and adherence remains the cornerstone of the HIV treatment, care and support programme. Counselling and group support are valuable support tools for HIV-positive AYAs.^27^

### Limitations

The study was limited to four public health institutions from one district in the province, hence the results might not be generalisable. The exclusion of ages below 18 and undocumented viral load were another limitation.

## Conclusion

This study highlights the multifaceted challenges faced by adolescents and young adults living with HIV in adhering to ART. Addressing these challenges requires a comprehensive approach that includes enhancing healthcare delivery systems, improving provider attitudes, and fostering supportive environments for AYAs. The findings also highlight the necessity for treatment literacy to promote effective treatment coping strategies and a better understanding of the benefits of compliance in viral load suppression. In addition, there is a need for the implementation of age-appropriate care models for better facilitation of routine visits and management, including the availability of trained staff who can effectively engage with this demographic.
